# Supplementary material for: A Humanized Yeast Phenomic Model of Deoxycytidine Kinase to Predict Genetic Buffering of Nucleoside Analog Cytotoxicity
Source: Genes (Basel). 2019 Sep 30;10(10):770. doi: 10.3390/genes10100770 (PMC6826991; doi:10.3390/genes10100770)
Supplement: Supplementary file 1 [file genes-10-00770-s001.zip › Additional_Files/Additional_File7_GOTermHeatmaps.zip!/Additional_File7_GOTermHeatmaps/endosome_membrane.pdf]

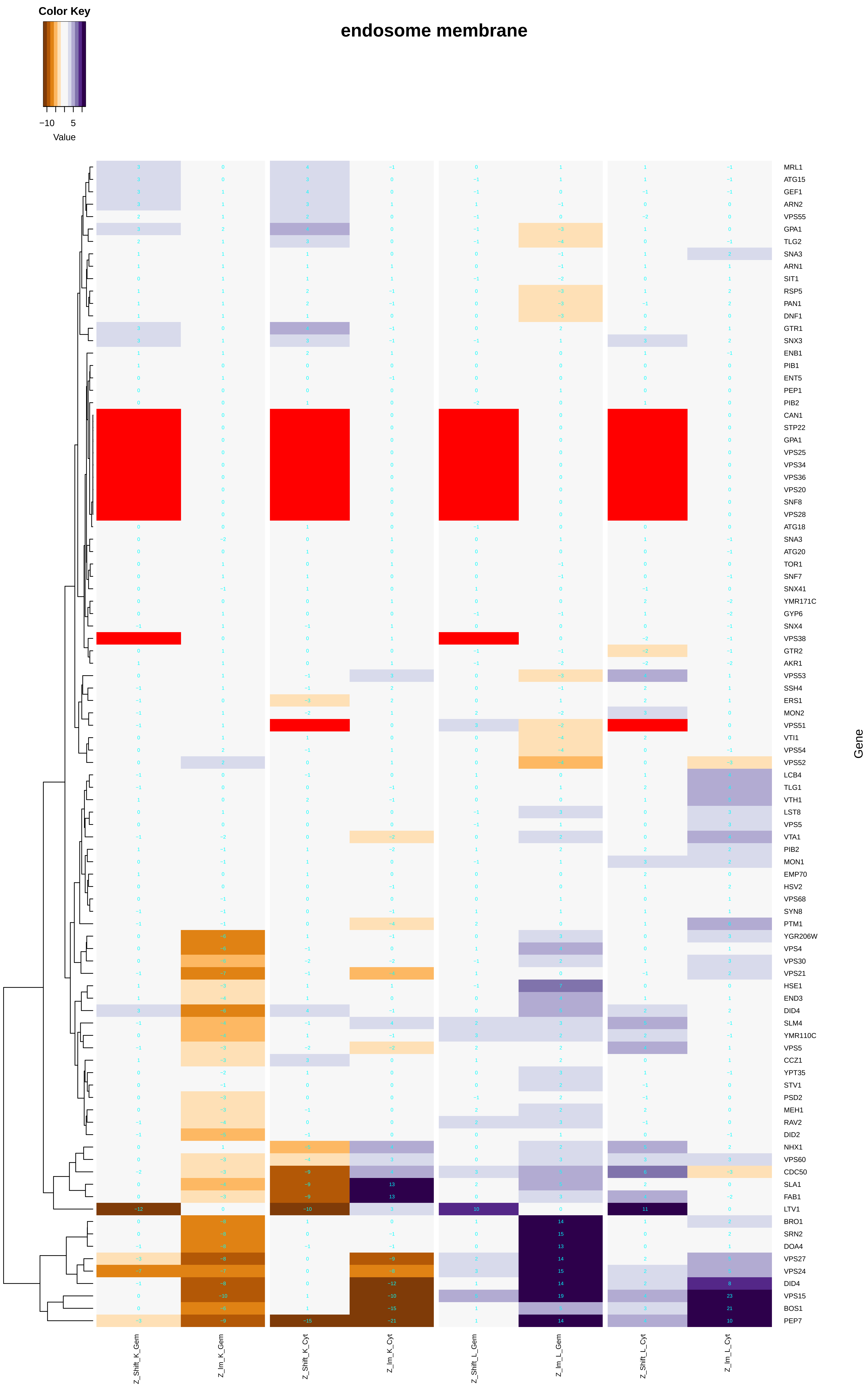

Color Key
−10
5
Value
endosome membrane
Gene
MRL1
ATG15
GEF1
ARN2
VPS55
GPA1
TLG2
SNA3
ARN1
SIT1
RSP5
PAN1
DNF1
GTR1
SNX3
ENB1
PIB1
ENT5
PEP1
PIB2
CAN1
STP22
GPA1
VPS25
VPS34
VPS36
VPS20
SNF8
VPS28
ATG18
SNA3
ATG20
TOR1
SNF7
SNX41
YMR171C
GYP6
SNX4
VPS38
GTR2
AKR1
VPS53
SSH4
ERS1
MON2
VPS51
VTI1
VPS54
VPS52
LCB4
TLG1
VTH1
LST8
VPS5
VTA1
PIB2
MON1
EMP70
HSV2
VPS68
SYN8
PTM1
YGR206W
VPS4
VPS30
VPS21
HSE1
END3
DID4
SLM4
YMR110C
VPS5
CCZ1
YPT35
STV1
PSD2
MEH1
RAV2
DID2
NHX1
VPS60
CDC50
SLA1
FAB1
LTV1
BRO1
SRN2
DOA4
VPS27
VPS24
DID4
VPS15
BOS1
PEP7
Z_Shift_K_Gem
Z_Im_K_Gem
Z_Shift_K_Cyt
Z_Im_K_Cyt
Z_Shift_L_Gem
Z_Im_L_Gem
Z_Shift_L_Cyt
Z_Im_L_Cyt

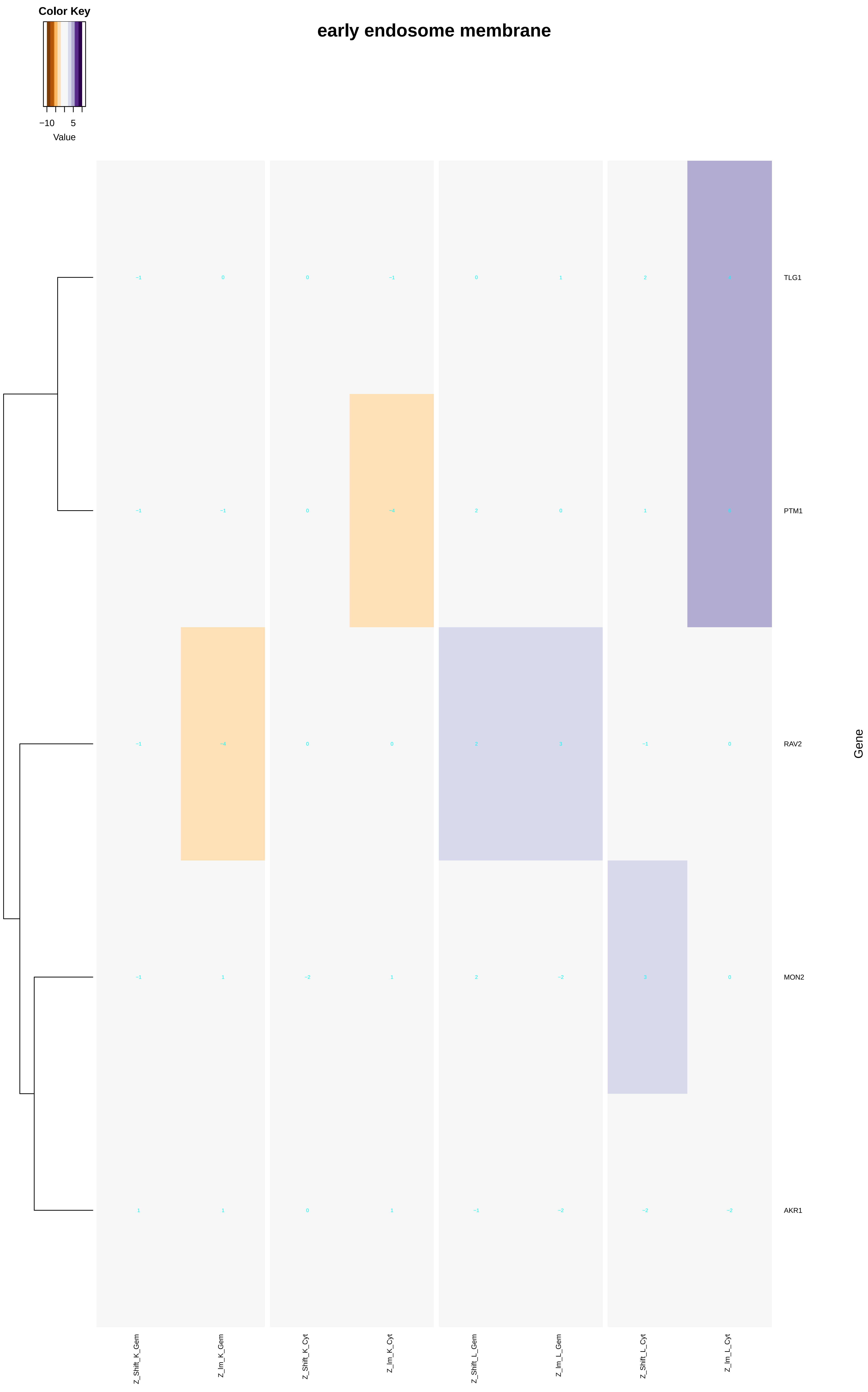

Color Key
−10
5
Value
early endosome membrane
TLG1
PTM1
RAV2
MON2
AKR1
Gene
Z_Shift_K_Gem
Z_Im_K_Gem
Z_Shift_K_Cyt
Z_Im_K_Cyt
Z_Shift_L_Gem
Z_Im_L_Gem
Z_Shift_L_Cyt
Z_Im_L_Cyt

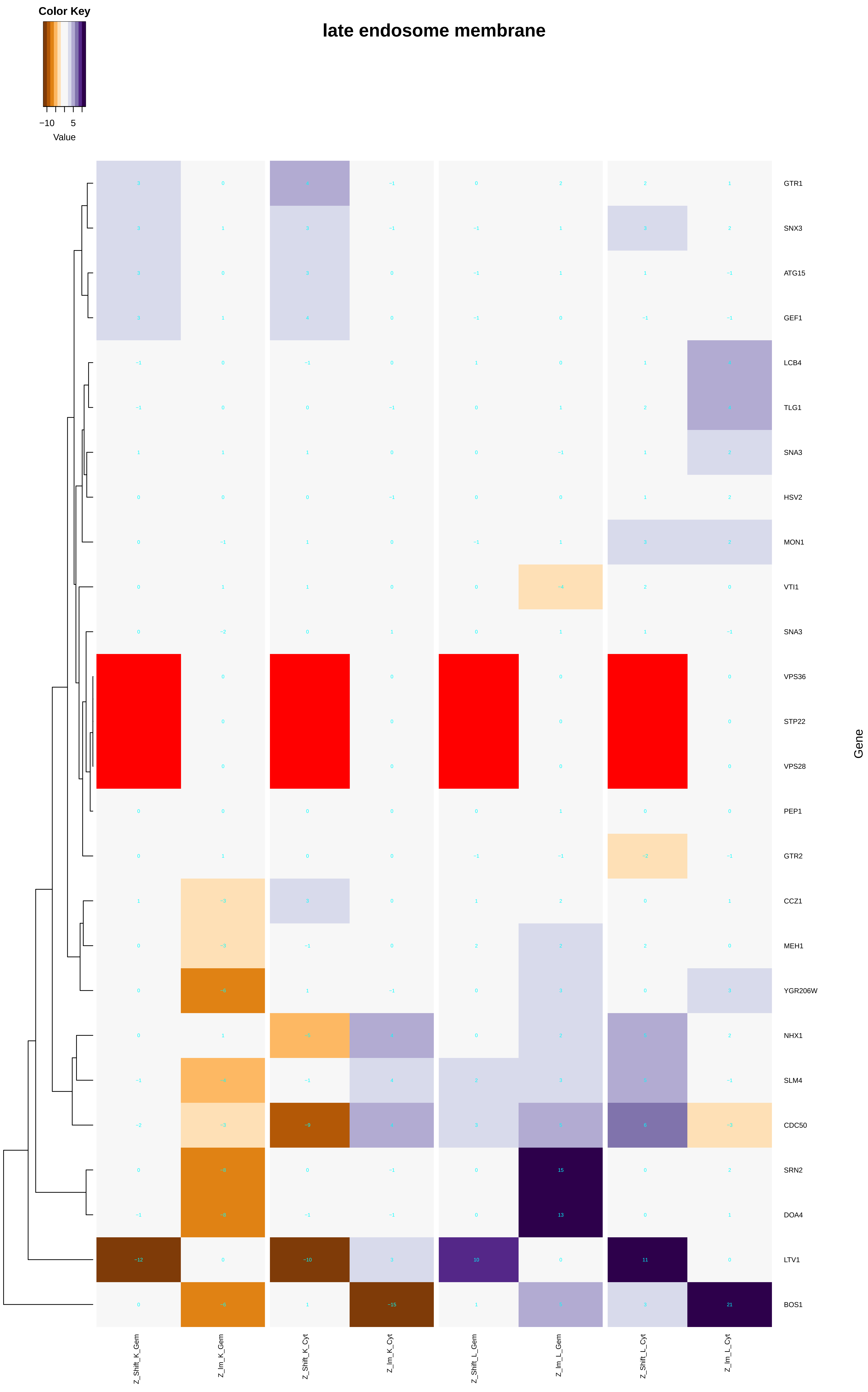

Color Key
−10
5
Value
late endosome membrane
GTR1
SNX3
ATG15
GEF1
LCB4
TLG1
SNA3
HSV2
MON1
VTI1
SNA3
VPS36
STP22
VPS28
PEP1
GTR2
CCZ1
MEH1
YGR206W
NHX1
SLM4
CDC50
SRN2
DOA4
LTV1
BOS1
Gene
Z_Shift_K_Gem
Z_Im_K_Gem
Z_Shift_K_Cyt
Z_Im_K_Cyt
Z_Shift_L_Gem
Z_Im_L_Gem
Z_Shift_L_Cyt
Z_Im_L_Cyt

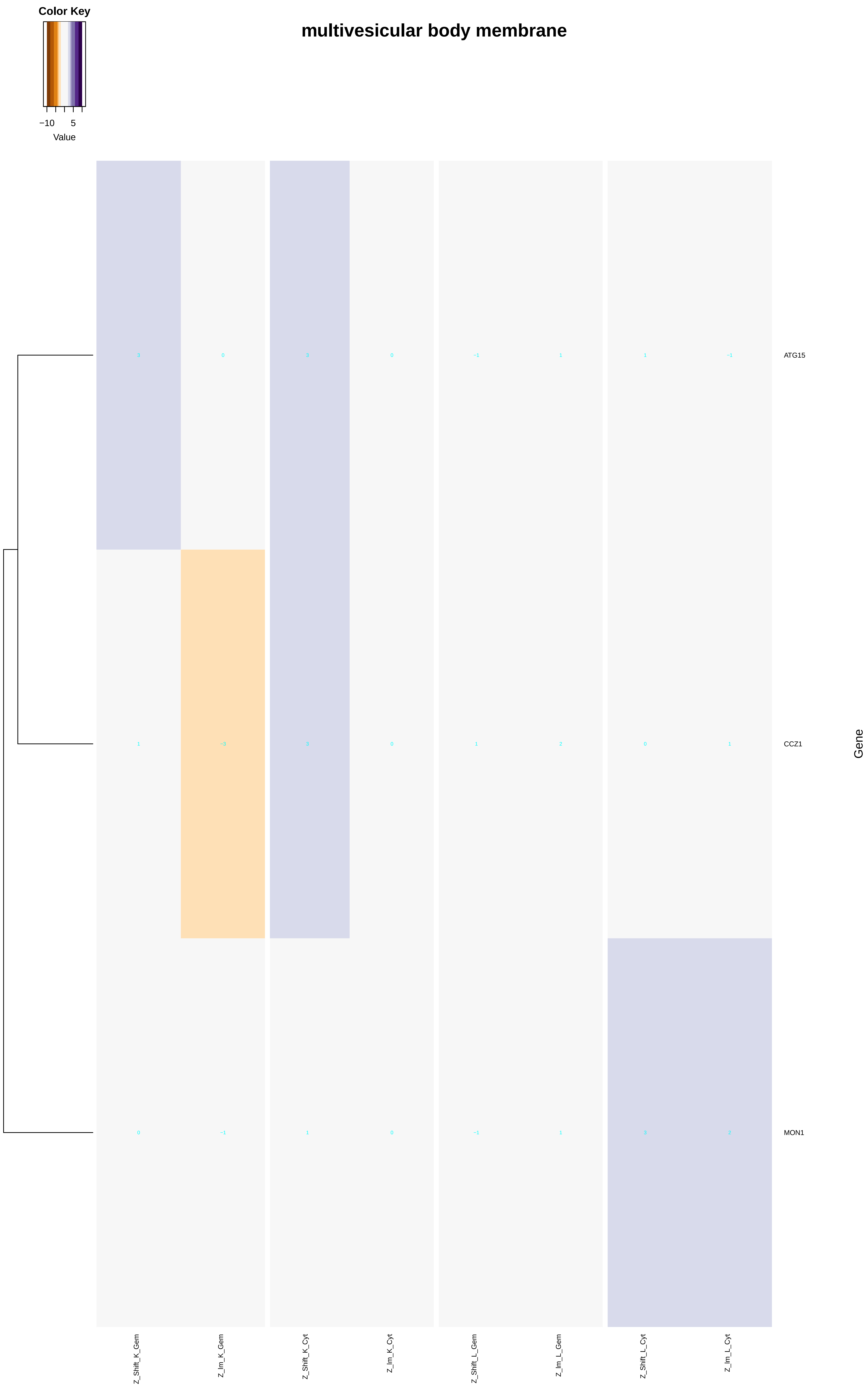
Color Key
−10
5
Value
multivesicular body membrane
ATG15
CCZ1
MON1
Gene
Z_Shift_K_Gem
Z_Im_K_Gem
Z_Shift_K_Cyt
Z_Im_K_Cyt
Z_Shift_L_Gem
Z_Im_L_Gem
Z_Shift_L_Cyt
Z_Im_L_Cyt
